# The cortical thickness phenotype of individuals with *DISC1* translocation resembles schizophrenia

**DOI:** 10.1172/JCI82636

**Published:** 2015-08-24

**Authors:** Orla M. Doyle, Catherine Bois, Pippa Thomson, Liana Romaniuk, Brandon Whitcher, Steven C.R. Williams, Federico E. Turkheimer, Hreinn Stefansson, Andrew M. McIntosh, Mitul A. Mehta, Stephen M. Lawrie

**Affiliations:** 1 Department of Neuroimaging, Institute of Psychiatry, Psychology and Neuroscience, King’s College London, London, United Kingdom.; 2 Division of Psychiatry, University of Edinburgh, Edinburgh, United Kingdom.; 3 Centre for Genomics and Experimental Medicine, Institute of Genetics and Molecular Medicine, University of Edinburgh, Western General Hospital, Edinburgh, United Kingdom.; 4 Clinical and Translational Imaging, Pfizer, Cambridge, Massachusetts, USA.; 5 deCODE Genetics/Amgen, Reykjavik, Iceland.

**Keywords:** Genetics, Neuroscience

## Abstract

**BACKGROUND.** The disrupted in schizophrenia 1 (*DISC1*) gene locus was originally identified in a Scottish pedigree with a high incidence of psychiatric disorders that is associated with a balanced t(1;11)(q42.1;q14.3) chromosomal translocation. Here, we investigated whether members of this family carrying the t(1;11)(q42.1;q14.3) translocation have a common brain-related phenotype and whether this phenotype is similar to that observed in schizophrenia (SCZ), using multivariate pattern recognition techniques.

**METHODS.** We measured cortical thickness, cortical surface area, subcortical volumes, and regional cerebral blood flow (rCBF) in healthy controls (HC) (*n* = 24), patients diagnosed with SCZ (*n* = 24), patients diagnosed with bipolar disorder (BP) (*n* = 19), and members of the original Scottish family (*n* = 30) who were either carriers (T+) or noncarriers (T–) of the *DISC1* translocation. Binary classification models were developed to assess the differences and similarities across groups.

**RESULTS.** Based on cortical thickness, 72% of the T– group were assigned to the HC group, 83% of the T+ group were assigned to the SCZ group, and 45% of the BP group were classified as belonging to the SCZ group, suggesting high specificity of this measurement in predicting brain-related phenotypes. Shared brain-related phenotypes between SCZ and T+ individuals were found for cortical thickness only. Finally, a classification accuracy of 73% was achieved when directly comparing the pattern of cortical thickness of T+ and T– individuals.

**CONCLUSION.** Together, the results of this study suggest that the *DISC1* translocation may increase the risk of psychiatric disorders in this pedigree by affecting neurostructural phenotypes such as cortical thickness.

**FUNDING.** This work was supported by the National Health Service Research Scotland, the Scottish Translational Medicine Research Collaboration, the Innovative Medicines Initiative (IMI), the Engineering and Physical Sciences Research Council (EPSRC), The Wellcome Trust, the National Institute of Health Research (NIHR), and Pfizer.

## Introduction

Schizophrenia (SCZ) can be a chronic, debilitating psychiatric disorder; it affects approximately 1% of the adult population (1). Neuroimaging has consistently demonstrated widespread structural and functional brain abnormalities (2–5). Unaffected family members and twins of patients, in some cases, show subtle abnormalities in brain structure and function (6), supporting the theory that genetic factors play a major role in the development of the disorder. Numerous genome-wide studies have identified regions of the genome that harbor risk genes for SCZ (7, 8). These effects are often small and can be difficult to interpret biologically; individually, their downstream effects may reveal relatively little about the mechanistic basis of SCZ. In contrast, rare, highly penetrant variants account for a small fraction of the liability — but their high correlation with the disease phenotype makes these mutations ideal as potential models of SCZ. The disrupted in schizophrenia 1 (*DISC1*) gene locus is a particular case. *DISC1* was originally identified in a large Scottish family (9), spanning the chromosome 1 breakpoint of balanced chromosomal translocation t(1;11)(q42.1;q14.3) (10). A majority of the family members with this chromosomal abnormality have a heavy burden of major psychiatric disorders, but no physical, neurological, or dysmorphic conditions (11). The involvement of the *DISC1* pathway in psychiatric disorders has since been reported in genetic association studies within different pedigrees and more diverse populations (12–17). *DISC1* continues to feature strongly when considering genome-wide associations in terms of gene networks or in combination with biological function (18–20). However, large-scale genome-wide approaches have not identified a clear association between *DISC1* locus variants and SCZ (7). Therefore, although still of considerable interest for psychiatry, the status of *DISC1* as a risk factor for SCZ has been debated (21, 22).

The most commonly reported neurobiological phenotypes in animal models with mutations at *DISC1* are enlarged ventricles (23–26), cortical thinning (23), reduced cortical volume (25), reduction in parvalbumin GABAergic neurons (24, 25), and behavioral changes that together are suggestive of SCZ-like phenotypes (23–26). The brain-related phenotype in humans has been studied in the context of SNPs within *DISC1*. Cannon et al. (27) found that haplotype blocks of SNP markers spanning the *DISC1* and translin-associated factor X genes were associated with reduced gray matter density in the prefrontal cortex as well as several quantitative endophenotypic traits previously observed to covary with SCZ. Brauns et al. (28) examined the effect of *DISC1* SNPs on brain structure and function in healthy volunteers. For a particular SNP, they reported reduced cortical thickness in the left supramarginal gyrus. Carless et al. (29) investigated the brain-related phenotype in a large pedigreed cohort. They reported that variations in *DISC1* were most significantly associated with cortical thinning in regions often implicated in psychiatric disorders (30–32). Callicott et al. (33) used functional neuroimaging to show that an interaction between *DISC1* and GABA signaling via SLC12A2 has demonstrable effects on adult human hippocampal area function in vivo.

Here, we investigated whether members of the original Scottish family carrying the *DISC1* translocation have a common brain-related phenotype. Based on studies in experimental animals and humans, we expected widespread reductions in cortical thickness in *DISC1* translocation carriers. Importantly, we investigated how similar the neurophenotype is to what we observe in patients with SCZ using multivariate pattern recognition techniques (34). This methodology enabled us to train a model to separate healthy controls (HC) and patients and to apply this model to translocation carriers and noncarriers.

## Results

### Participants.

Following quality control of the imaging data, 5 participants from the group of patients diagnosed with SCZ and 1 participant from the group of HC were excluded. Therefore, 24 patients from the SCZ group had complete quality controlled data. We manually selected 24 participants from the HC group to match the 24 patients in the SCZ group for age and sex in a pairwise fashion (Table 1). For the family cohort of translocation carriers (T+) (*n* = 12) and noncarriers (T–) (*n* = 18), all 30 participants’ data passed quality control. Similarly, all data from patients recruited with bipolar disorder type I (BP) passed quality control.

### DISC1 translocation and SCZ.

Each participant in the T+ group had a psychiatric diagnosis with varying degrees of severity and a range of DSM-IV categories (Table 2). Diagnoses included major depression, psychosis phenotypes (SCZ and schizoaffective), and bipolar phenotypes (BP and cyclothymia). Three of the 18 family members without the translocation had a history of depression, but none were suffering from a current mental disorder.

**Figure 1:**
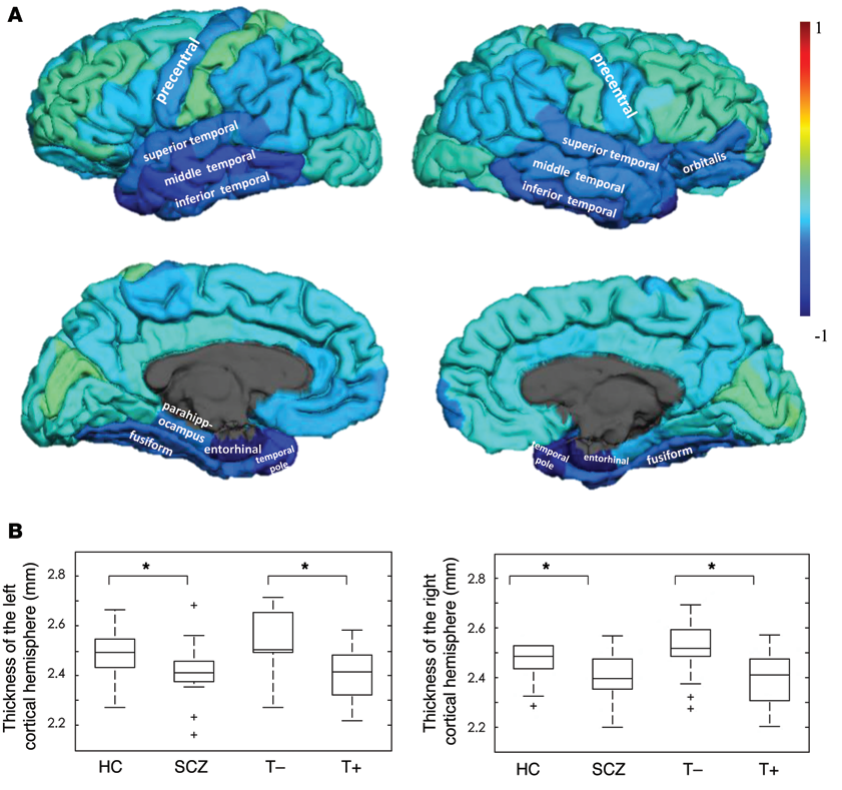
Pattern of cortical thickness across the groups. (**A**) Surface-based weight map showing the multivariate weights for the model discriminating between the HC (label –1) and the patients diagnosed with SCZ (label +1). (**B**) Box plot displaying cortical thickness across both hemispheres and across the 4 groups. *P < 0.05. Note that all 4 groups were age and sex matched for this comparison.

Statistically significant performance was found for 3 of the imaging measures: cortical surface area (AUC, 0.71; accuracy, 66.7%; *P* < 0.005), cortical thickness (AUC, 0.70; accuracy, 68.8%; *P* < 0.005 by permutation), and regional cerebral blood flow (rCBF) (AUC, 0.82; accuracy, 77.1%; *P* < 0.001). The model trained on the subcortical volumes did not reach significance. No statistically significant correlation was observed between the predictive probabilities of belonging to the SCZ group and dose of antipsychotic medication, as estimated by the chlorpromazine equivalents. The brain regions driving the model are visualized as weights displayed on the cortical surface in Figure 1A. These weights are all less than zero, which suggests widespread thinning of the cortex in the SCZ group. This is confirmed by data shown in Figure 1B, where we show that cortical thickness in both the left and right hemispheres is significantly reduced in the SCZ group when compared with the HC group (all 4 groups were matched for age and sex). Qualitatively, we note that the temporal lobe and precentral gyrus are strongly weighted regions.

**Figure 2:**
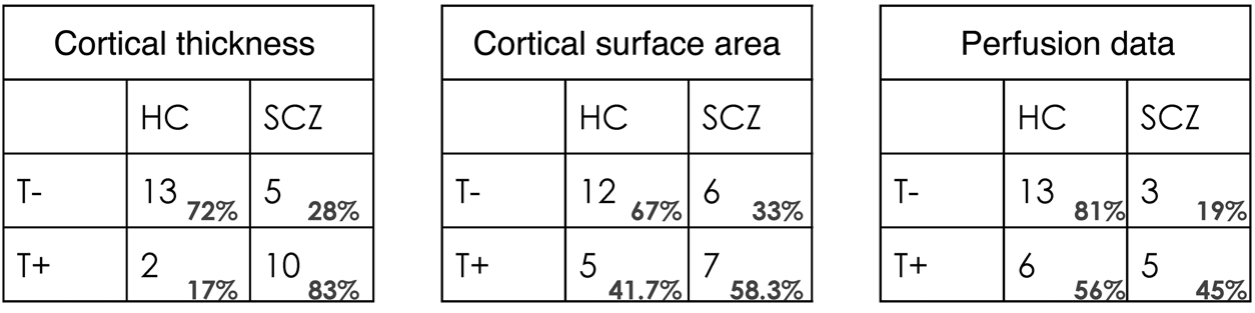
Prediction of belonging to the SCZ group across modalities. Contingency table for the family groups with and without the translocation (T+ and T–, respectively) tested using a classifier trained to discriminate between HC and patients diagnosed with SCZ. The rows represent the ground truth, i.e., T+ or T–, and the columns represent the predictions made by the model in relation to the HC versus SCZ contrast.

The translocation family members were then used as a test set for the 3 successful models (see Table 1 for the subject characteristics of the family members). The results are displayed as contingency tables in Figure 2. For cortical thickness, 72.2% (13/18) of the T– group were classified as belonging to the HC group and 83.3% (10/12) of the T+ group were classified as belonging to the SCZ group (*P* < 0.01). For cortical surface area, 66.7% (12/18) of the T– group were classified as belonging to the HC group and 58% (7/12) of the T+ group were classified as belonging to the SCZ group (*P* > 0.05). For the rCBF, 81% (13/16) of T– group were classified as belonging to the HC group and 45% (5/11) of the T+ group were classified as belonging to the SCZ group (*P* > 0.05).

**Figure 3:**
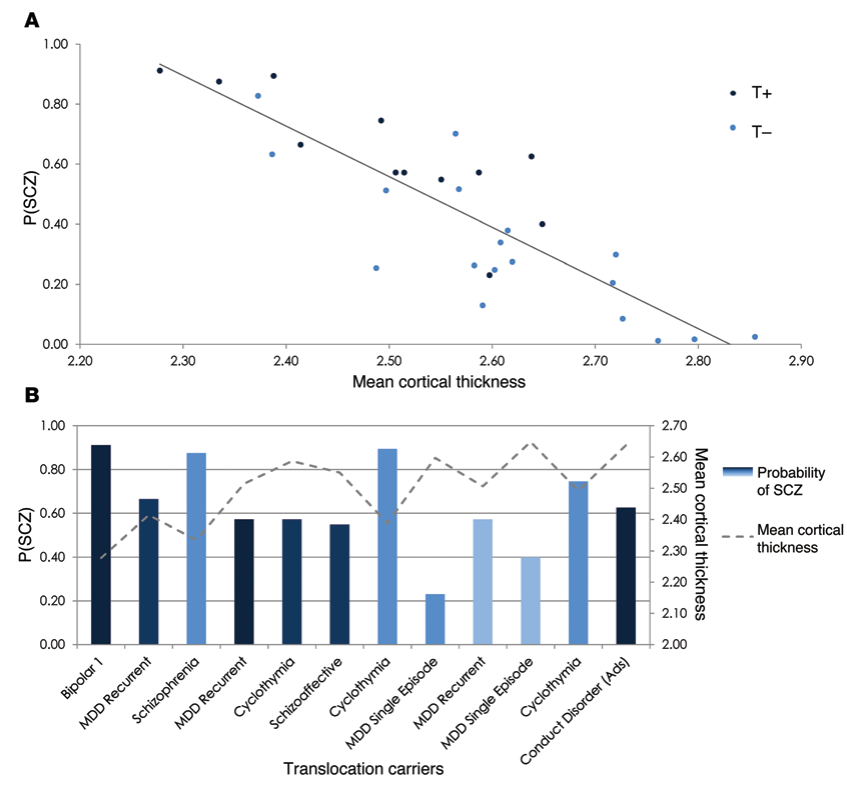
P(SCZ) from the cortical thickness classifier for the family cohort and the mean cortical thickness. (**A**) Mean cortical thickness plotted against P(SCZ) with the translocation carriers displayed in dark blue and the translocation noncarriers displayed in light blue. (**B**) The translocation carrier group displayed on an individual basis in terms of clinical diagnosis, P(SCZ), and mean cortical thickness. Conduct disorder (Ads), conduct disorder diagnosed as an adolescent.

In Figure 3A, the predictive probabilities of belonging to the SCZ group (P[SCZ]) are plotted against the mean cortical thickness. A strong negative correlation can be observed (Pearson’s rho, –0.86; *P* < 0.00001), indicating that reduced cortical thickness is associated with an increased probability of belonging to the SCZ group and, as in Figure 1B, we observed lower cortical thickness in the carriers. In Figure 3B, the mean cortical thickness, P(SCZ), and clinical diagnosis are displayed on an individual basis for the T+ group.

The correlation coefficient (Pearson’s rho) between the PANSS General Psychopathology scale (35) of the predictive probabilities was 0.52 (*P* = 0.0149) and following the exclusion of outliers using the Cook distance (36) was 0.45 (*P* = 0.0149). Considering the translocation positive group only, the correlation coefficient was 0.57 (*P* = 0.052) and following the exclusion of outliers was 0.47 (*P* = 0.14). The predictive probabilities of belonging to the SCZ group were not correlated with age or the estimated total intracranial volume.

### DISC1 translocation carriers versus noncarriers.

The T– and T+ groups were then compared directly. As the data are now used to train the model, we selected 14 participants from the T– group to match the T+ group for age and sex. The participants formed the group known as T–ʹ. The classification performance can be seen in Table 3. For cortical thickness, an AUC of 0.75 and corresponding accuracy of 73% were achieved, with significance at *P* < 0.01.

### Specificity of the cortical thickness phenotype.

To assess the model of cortical thickness in terms of its disease specificity, we tested the HC versus SCZ on a group of patients diagnosed with BP (age, 43.4 ± 13.2 years; sex, 13 M, 7 F). We found that 45% of bipolar patients were classified as belonging to the SCZ group and 55% were classified as belonging to the HC group (*P* > 0.05).

## Discussion

Here, we explore the brain-related phenotype of a balanced translocation at *DISC1* in the original Scottish family in which this genetic mutation was discovered. We focused on measures of brain structure and perfusion — cortical surface area, cortical thickness, subcortical volumes, and rCBF. Using 2 complimentary analyses, we found that cortical thickness was significantly associated with a balanced translocation at *DISC1*. First, members of the same family with and without the *DISC1* translocation could be discriminated based on cortical thickness. Second, the patients diagnosed with SCZ and those carrying the *DISC1* translocation shared a similar pattern of cortical thickness despite the heterogeneous clinical phenotype observed in the carriers, which included bipolar phenotypes, recurrent depression, and SCZ. This pleiotropic property of variation at DISC1 has been widely reported in other studies as well as within the pedigree studied in this work (10, 11). The cortical surface area and rCBF were not sensitive to translocation at *DISC1*, suggesting that the cortical thickness phenotype represents a more specific feature that is shared by SCZ and variation at DISC1.

We observed widespread reductions in cortical thickness in the SCZ and T+ groups (Figure 1; all 4 groups were matched for age and sex) in a pattern similar to that reported by Carless et al. (29) related to common variants in the *DISC1* genotype. Reductions in regional cortical thickness have been reported in psychiatric conditions linked to *DISC1*, namely SCZ (37, 38), BP (32), and major depressive disorder (MDD) (39). Studies investigating subjects at ultrahigh risk (30) and those whose relatives suffered from either depression (32) or SCZ (40) also report changes in cortical thickness despite the absence of the clinical phenotype. Translocation carriers were also found to have significantly reduced P300 amplitude and latency in a manner similar to subjects with SCZ but distinct from that of family members who do not carry the translocation and control subjects (11). Therefore, carriers were found to resemble patients diagnosed with SCZ even though several of the carriers had no psychiatric diagnosis. This indicates that inheritance of the translocation results in consistent disturbances in brain function, which may not always cosegregate with psychiatric diagnosis. As an example, we found that the discriminative model of cortical thickness for HC versus SCZ was not sensitive to the BP in a clinical cohort. However, the subset of translocation carriers with bipolar phenotypes was indeed classified as belonging to the SCZ group (see Figure 3B) and the 2 carriers who were classified as HC both had a clinical diagnosis of single-episode MDD. These findings together with our data support the hypothesis that variation in *DISC1* affects the normal spectrum of brain development and function, producing some features that are redolent of SCZ but are not representative of a phenocopy of the disorder. Furthermore, our data indicate that the consistency in the cortical thickness phenotype of translocation at DISC1 does not simply translate to a consistent clinical phenotype, which is likely to be dependent on other factors, whether genetic and/or environmental.

The role of *DISC1* has also been studied using animal models of disruption at this locus. Transgenic *DISC1* mice have been developed using different approaches and experimental paradigms (23–26). These studies reported enlarged lateral ventricles (23, 24, 26), reduced cerebral cortex (25) and the thinning of layers II/III (23), reduction in parvalbumin neurons (23, 24), and attenuation of neurite outgrowth in the cortex (23, 26) as well as behavioral changes. Ayhan et al. reported that a mouse model of mutant *DISC1* has differential effects across various stages of neurodevelopment (prenatal, postnatal, and both) (25). These studies have reported consistent findings indicating that mutation at *DISC1* results in neuropathological and behavioral changes that are reminiscent of (but not limited to) the findings in major psychiatric disorders, in particular, SCZ (41). Overall, these studies add to the accumulating evidence that DISC1 plays an important role in cortical genesis. To date, these animal studies have provided qualitative links between variations at *DISC1* and SCZ. Our work represents one of the first studies to quantitatively compare the abnormalities in brain structure and function due to variation at *DISC1* to those abnormalities associated with SCZ. We confirm that the pattern of cortical thinning observed in carriers of the translocation is highly similar to that observed in the SCZ group, whereas rCBF and cortical surface area do not share a common pattern of abnormalities.

One caveat of this study is the possible effect of medication. For the SCZ group, all but 5 subjects were medicated at the time of scanning. Given that the patients in the SCZ group were receiving different combinations of medications (mood stabilizers, antipsychotics, antidepressants, etc.) that could have differential effects on the brain, we do not expect the effect of medication to have driven the classification between HC and SCZ. Certainly, the predictive probabilities of belonging to the SCZ group did not correlate with the level of medication, as measured by chlorpromazine equivalents. Finally, members of the translocation carrier group that were identified as having a cortical thickness phenotype similar to that of the SCZ group were mostly medication free, with only 2 subjects medicated with both mood stabilizers and antipsychotics.

Another consideration is the specificity of the model trained to discriminate between the HC and SCZ groups. It is possible that the classification may be driven by nonspecific features related to comparing healthy with nonhealthy participants. However, for the model to perform with high sensitivity (i.e., accurate classification of those with SCZ), a consistent pattern of brain changes would need to be identified in the SCZ group; as this is the case reported here, we conclude that a shared pattern of brain changes is present in the SCZ group. To assess the specificity of the cortical thickness phenotype, we tested the HC versus SCZ in a group of patients diagnosed with BP. The BP group was randomly assigned to the HC and SCZ group, implying that the classifier is not merely a model of disease versus health but is specific to the pattern of cortical thinning in SCZ. We speculate that this may be because patients with BP have a lesser developmental component to their adult brain structure. A longitudinal study would be required to fully realize the role of development in the cortical thickness phenotype we demonstrate here. It would also be interesting to understand the results presented here in the context of MDD, which has been diagnosed in 5 of the T+ group (3 of which have a recurrent diagnosis) and 3 of the T– group (1 recurrent). These data could enable a richer description of the brain-related phenotype of variation at *DISC1*.

The sample size for the family is modest, which is to be expected given that we were limited to members of the original Scottish family. We believe that the uniqueness of this cohort serves to compensate for the modest sample size. Importantly, our methodology enables us to test individual family members using models trained with either the HC and SCZ groups or the family data directly.

Although implicated as a risk factor for psychiatric disorders, a consensus on the role of the *DISC1* gene has not yet been reached. Here, we identified that the pattern of cortical thinning, but not rCBF, cortical surface area, or subcortical volumes, is a robust phenotype for translocation at *DISC1*. The pattern of cortical thinning is also highly similar to that observed in patients diagnosed with SCZ. The biological roles of *DISC1* fit well with our current knowledge on the etiological roots of SCZ. Our data suggests that the t(1:11) translocation in this pedigree could increase the risk of psychiatric disorders including SCZ through affecting neurostructural phenotypes such as cortical thickness.

## Methods

### Participants.

Twenty-nine patients diagnosed with SCZ and 19 patients diagnosed with BP were recruited. Forty-one HC were recruited. Thirty participants were recruited from a previously reported Scottish family (9–11). Karyotyping using a custom PCR method (42, 43) was used to identify the presence of the translocation in 12 family members (translocation-positive group [T+]) and absence in 18 family members (translocation-negative group [T–]).

### PCR typing of translocation breakpoint.

The translocation status of all participants was tested on new blood samples using PCR-based methods. Primers were designed to span the t(1;11) breakpoint (44), using the Primer 3 primer design program (42, 43). The translocation primers were as follows: t1;11_chr1TTTCTTTGGAAGGCACCTTATC, t1;11_Chr11AGCAAAGTGGGTGAAGAATAGAG (PCR product size, 1105 bp). DNA was coamplified in the same reaction using DISC1 exon 9 primers (DISC1, Ex9f TTCCCCAGAGGACTGCTAAG; DISC1, Ex9r AAATGTCCCCAAGGAAAAGG, PCR product size, 484 bp) to verify the assay function. PCR was performed in a total volume of 10 μl with 20 ng DNA, 1× reaction buffer with 1.5 mM MgCl2 (PerkinElmer), 100 μM of each dNTP (Peqlab), 0.5 U Taq DNA polymerase (Sigma-Aldrich), and 0.33 μM of each primer (Sigma-Aldrich). PCR cycling was carried out on a PTC-225 thermal cycler (MJ Research). PCR cycling conditions consisted of denaturation at 95°C for 1 minute, followed by 10 cycles of 93°C for 20 seconds, 70°C for 30 seconds, minus 1°C/cycle and 72°C for 1 minute, followed by 30 cycles of 93°C for 20 seconds, 60°C for 30 seconds and 72°C for 1 minute, and a final extension of 72°C for 10 minutes. Five microliters of the PCR product was resolved on a 1.5% agarose gel, and PCR product size was estimated against 250 ng of λ HindIII size standard (Life Technologies).

### Clinical assessment.

Clinical diagnoses of all participants were established during face-to-face interviews conducted by a consultant psychiatrist using the structured clinical interview for DSM-IV (SCID) (45). Life-time diagnoses, according to DSM-IV criteria, were reached by consensus between 2 psychiatrists. If there was any disagreement in diagnosis, then this was resolved on discussion with the senior clinical researcher (S.M. Lawrie). These diagnoses were based on the interviews and supplemented, where appropriate, by inspection of hospital case records, collateral information from relatives and carers, and clinical data collected at the time of previous follow-up contacts. Participants were further assessed using the PANSS questionnaires (46). Symptom ratings took place within 1 week of the MRI acquisition.

### Neuroimaging.

Brain imaging was performed at the Clinical Research Imaging Centre (http://www.cric.ed.ac.uk/). The radiographer who performed the acquisition was not made aware of the clinical or genetic status of the participants. Imaging was performed on a Siemens Verio 3T scanner using the matrix head coil with 12 elements. A structural brain image was acquired using a T1-weighted magnetization prepared rapid acquisition gradient echo sequence prescribed parallel to the AC-PC line with repetition time (TR) = 2300 ms, echo time (TE) = 2.98 ms, inversion time (TI) = 900 ms, and flip angle = 9°, yielding 160 contiguous 1-mm slices of 256 × 256 voxels. Perfusion imaging was performed to quantify rCBF using the PICORE Q2TIPS pulsed arterial spin labeling sequence (22 slices, TR = 3000 ms, TE = 14 ms, TI1 = 700 ms, T1-stop = 1400 ms, and TI2 = 1600 ms).

### Preprocessing.

All scans were anonymized at the time of acquisition. Image preprocessing followed a set protocol, and the analysis protocol was strictly adhered to regardless of the genetic or clinical status of the participant. Structural images were processed using FreeSurfer v5.3 (http://surfer.nmr.mgh.harvard.edu/). Volumetric segmentation, cortical surface reconstruction, and cortical parcellation were used to quantify the thickness and surface areas of cortical anatomical regions. Cortical anatomical regions were defined by the Desikan-Killiany atlas (47). Subcortical volumes were defined as by Fischl and colleagues (48). Cortical surface area and the subcortical volumes were normalized by the total intracranial volume for each subject (49).

Perfusion images were preprocessed using ASLtbx (50). Briefly, motion correction was performed on the perfusion time series using 6-parameter rigid body spatial transformation. The perfusion images were then coregistered to each subject’s structural image. The images were then smoothed using a Gaussian kernel, and a brain mask was generated. CBF quantification was performed to produce a mean CBF image per subject. The CBF maps were then spatially normalized into a standard space using the MNI 125 averaged brain template. Full brain coverage was not acquired for each subject; therefore, after preprocessing, a consensus mask of voxels was created and applied to all images so that the same number of voxels was considered for each subject. Each image was scaled by the mean across all voxels.

### Multivariate pattern classification.

Multivariate pattern classification can be used to discriminate between groups of subjects by modeling the spatial pattern of brain changes (51, 52). This is particularly advantageous for detecting neurobiological changes associated with psychiatric disorders, which are often subtle and widespread across the brain, i.e., inherently multivariate. Moreover, the classifier can be trained to distinguish between HC and SCZ and then used to assign a probability of belonging to the SCZ group for independent participant data. This reflects how similar the underlying pattern of neurobiology for the T– and T+ groups is to that of either SCZ or HC. To assess the specificity of the HC versus SCZ model, we used the BP group as a test set.

Classification aims to learn the mapping between a multivariate data source (e.g., cortical thickness) and a label of interest (+1 or –1; e.g., belonging to the T+ or T– group) in order to predict the label for a new participant. An important consideration for these approaches is “overfitting,” whereby the model is overly complex and does not generalize well to new, unseen data. To help alleviate this, we used approaches that incorporate regularization that penalizes model complexity. Moreover, pattern recognition was performed using crossvalidation whereby a portion of the data was preserved for independently testing the model.

We employed Gaussian process classification (GPC) implemented in a Bayesian framework. GPC has been well validated in the neuroimaging community across a wide range of applications, including psychiatry (53–56) and neurology (57–59). We focused on this approach rather than the commonly used support vector machines (60), as GPC provides similar performance and also probabilistic estimates of class membership quantifying the uncertainty of the individual predictions. For a detailed overview of this approach, we refer the reader to Rasmussen and Williams (61). GPC was implemented using a linear model within the GPML v3.1 toolbox (http://www.gaussianprocess.org/gpml/code/matlab/doc/) (62).

**Figure 4:**
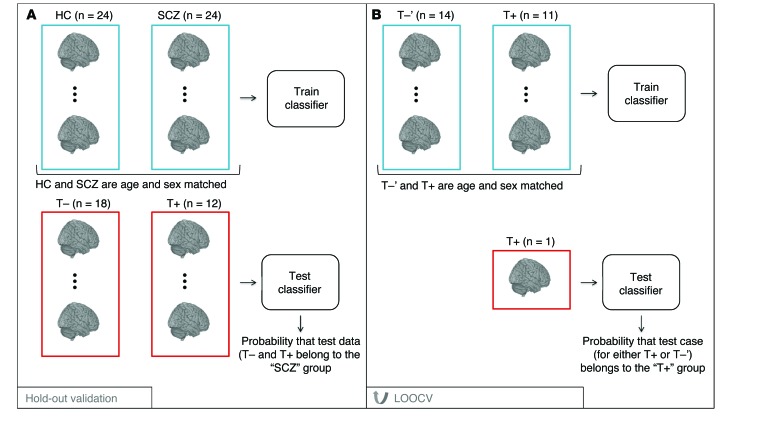
Pipeline for the 2 machine-learning schemes used in this work. (**A**) The classifier is trained to discriminate between HC and patients diagnosed with SCZ (matched for age and sex). The trained classifier is then tested on the members of the Scottish family who do not carry the translocation (T–) and those that do carry the translocation (T+). (**B**) The classifier is trained in LOOCV to discriminate between age- and sex-matched participants from the T–ʹ and T+ groups.

Classification was performed to answer the following questions: (a) how similar are those in the T+ group to those in the SCZ group in terms of their brain-related phenotype (see Figure 4A), and (b) do the T+ group members have a distinct brain-related phenotype in comparison with those in the T– group (see Figure 4B)? First, we developed a model that discriminates between the HC and SCZ groups. To prevent nuisance covariates from driving the classification, we ensured that the training groups were matched for age and sex. We assumed that test samples were independent and therefore matching across test groups was not required. Nonetheless, the ages of the test samples were in the same range as the ages of the training sample, and covarying for age across training and test data did not alter the findings we present. The model was trained using pairwise leave-one-out crossvalidation (pLOOCV) on the HC versus SCZ data whereby all but 1 pair of matched subjects’ data were used to train the model and the pair of subjects left out was used as the test case; this process was iterated until each pair of subjects had served as a test case.

To assess whether the classification accuracy for a particular contrast was significantly greater than chance (50%), we used permutation testing. To achieve this, the training labels used for classification were shuffled (permuted), the classifier was trained using these permuted labels, and then the trained model was applied to the test data. The accuracy of the classifier in detecting the true test labels based on permuted labels is known as the permuted accuracy. The procedure of permuting the training labels was repeated 1,000 times to produce the null distribution of accuracies. We then counted the number of times that the permuted accuracy was greater than that obtained using the true training labels. This count was divided by the number of permutations (1,000) to estimate a *P* value for the classification accuracies.

When statistically significant, the model was trained on all of the HC versus SCZ data and tested on the translocation groups to provide a prediction of which group (HC or SCZ) the data from the family (T+ and T-) are most similar to. To assess whether the predictions for the T+ and T– groups were significantly different from chance, we used Fisher’s exact test. We expected the T– cases to belong to the HC group and the T+ cases to belong to the SCZ group. As stated above, it was crucial to match the training data for nuisance covariates and then assume that the test data were drawn from the same range. Nonetheless, to robustly assess the effect of age, the correlation (Pearson’s correlation) between predictive probabilities of belonging to the SCZ group with the age of the family members was computed. For the family, we also assessed the correlation between the predictive probabilities of belonging to the SCZ group and the PANSS General Psychopathology scale. As the sample size investigated here is modest, we used the Cook distance measure to assess whether potential correlations were driven by outliers in the data (36). We excluded 3 subjects with a Cook distance greater than 0.5 (63). If subjects reached the exclusion threshold, they were removed and the correlations were recalculated.

Second, the model was trained on the T– versus T+ data directly using LOOCV (see Figure 4B). As the family data were now used to train the model, the T– group was downsampled to match the T+ group for age and sex; we refer to this group as T–ʹ. The LOOCV procedure was implemented rather than pLOOCV, as the T+ and T–ʹ groups were not equally sized and hence the paired approach was not appropriate. To assess the relationship between the certainty of the predictions and the dose of medication, we tested the correlation (Pearson’s correlation) between the predictive probabilities of belonging to SCZ group and the dose of antipsychotic medication. To visualize the pattern of brain regions driving the discriminative model, we mapped the multivariate weight patterns. These weight patterns are sensitive to the spatial covariance in the data; therefore, we avoided performing local statistical inference and instead considered the pattern. Note that the weights extracted from the Gaussian process classifier do not correspond directly to magnitude changes across groups, as they are influenced by additional factors such as variance and covariance.

### Statistics.

Permutation testing was used to compute the *P* value for a particular classification accuracy. To investigate the linear relationship between variables, Pearson’s product-moment correlation coefficient was computed and the associated *P* value was assessed for significance. The Wilcoxon signed-rank test was used to assess differences in cortical thickness across groups. In all cases, *P* values of less than 0.05 were considered significant.

### Study approval.

This study was approved by the Multi-Centre Research Ethics Committee in Scotland. All study participants gave their written, informed consent.

## Figures and Tables

**Table 3:**
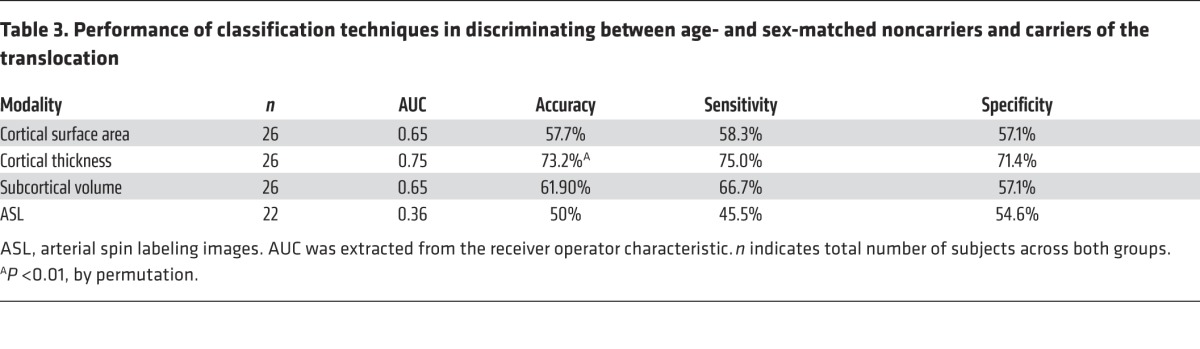
Performance of classification techniques in discriminating between age- and sex-matched noncarriers and carriers of the translocation

**Table 2:**
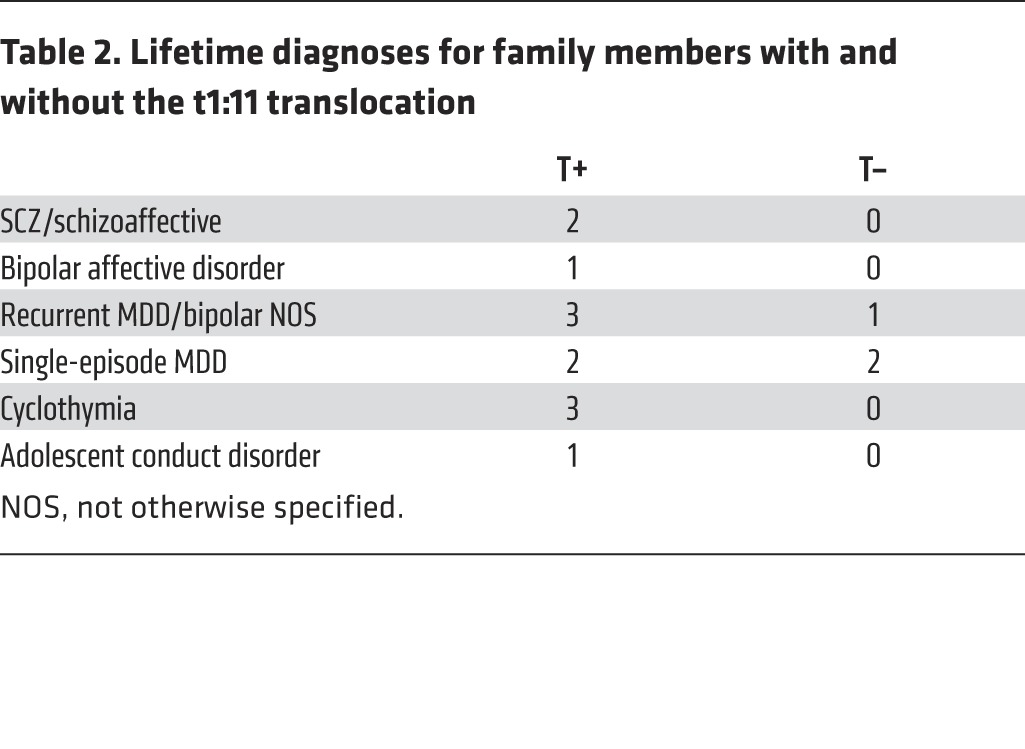
Lifetime diagnoses for family members with and without the t1:11 translocation

**Table 1:**
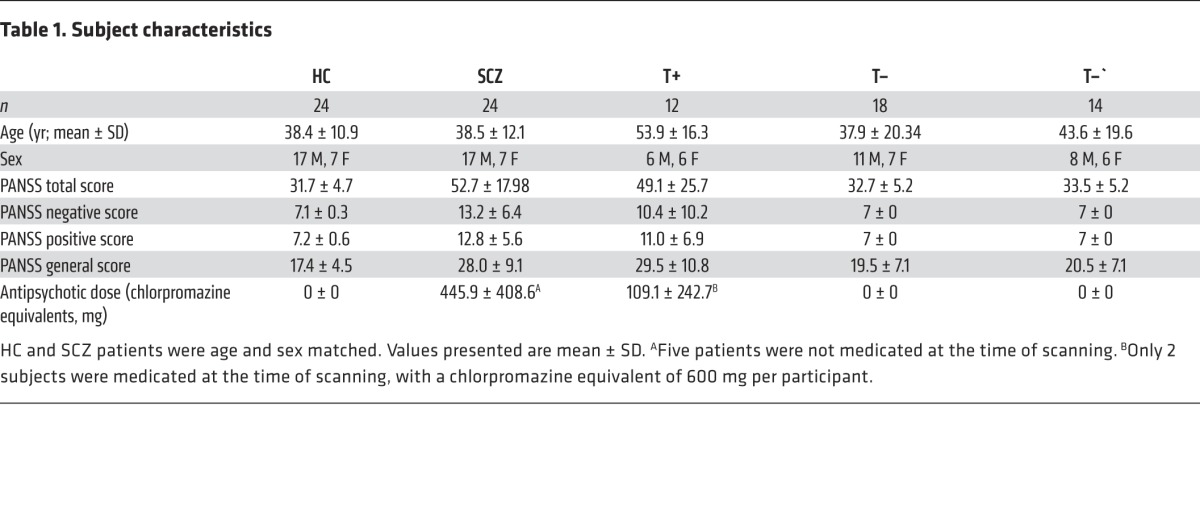
Subject characteristics
